# Establishment of a Mutant Library for Infection Cushion Development and Identification of a Key Regulatory Gene in *Botrytis cinerea*

**DOI:** 10.3390/jof11010016

**Published:** 2024-12-29

**Authors:** Maoyao Tang, Kexin Wang, Pan Zhang, Jie Hou, Xiaoqian Yu, Hongfu Wang, Yangyizhou Wang, Guihua Li

**Affiliations:** 1 College of Plant Sciences, Jilin University, Changchun 130062, China; tangmy21@mails.jlu.edu.cn (M.T.); kxwang22@mails.jlu.edu.cn (K.W.); xqyu23@mails.jlu.edu.cn (X.Y.); whf24@mails.jlu.edu.cn (H.W.); wyyz17602868149@163.com (Y.W.); 2 Engineering Research Centre of Forestry Biotechnology, Jilin Province in Beihua University, Jilin 132013, China; zp13944197374@163.com (P.Z.); nihaojiehou@163.com (J.H.)

**Keywords:** forward genetics, mutant library, ATMT, *EXO70*

## Abstract

*Botrytis cinerea*, the grey mould fungus affecting over 1400 plant species, employs infection cushion (IC), a branched and claw-like structure formed by mycelia, as a critical strategy to breach host surface barriers. However, the molecular mechanisms underlying IC formation remain largely unexplored. In this study, we utilized a forward genetics approach to establish a large T-DNA tagged population of *B. cinerea*, which contained 14,000 transformants. Through phenotype screening, we identified 161 mutants with defects in IC development. Detailed analyses revealed that these mutants exhibited various degrees of impairment in IC formation, ranging from complete failure to form ICs to a reduction in the number and maturity of ICs. Further genetic analysis of one of the mutants led to the identification of *EXO70*, a gene encoding a component of the exocyst complex, as a key regulatory factor in IC development. Mutants with deletion of *EXO70* failed to form ICs, confirming its crucial role in the process. The mutant library reported here provides a rich resource for further large-scale identification of genes involved in IC development. Our findings provide valuable insights into the genetic and molecular basis of IC formation and offer new targets for controlling *B. cinerea* pathogenicity.

## 1. Introduction

*Botrytis cinerea* infects over 1400 plant species, causing grey mould disease, which severely threatens agricultural production [1,2,3]. The surface of host plants has powerful physical barriers, including wax, cuticle, and cell wall, which effectively block the invasion of most pathogens. As a notorious and successful crop killer, *B. cinerea* has evolved strategies to break through these barriers, one of which is the infection cushion (IC), a branched and claw-like structure, with the infectious tip formed by clusters of short, bead-like cells [4,5]. By forming this large, sometimes exaggerated, infection structure, *B. cinerea* can breach most of the host’s physical barriers and invade the plant, creating opportunities for further parasitic establishment.

*B. cinerea* primarily uses conidia as the source of primary and secondary infections, which are spread via wind, rain, irrigation, or agricultural activities [6]. Upon reaching the plant surface, conidium germinate and form a germ tube under cool and high-humidity conditions. The tip of the germ tube is induced by host surface signals to develop into appressorium or further branch out to form a more complex and powerful IC for host penetration. Germ tubes can also invade through decaying flowers, wounds, or necrotic tissues [4]. Inoculation experiments have shown that when conidial concentrations are as low as 2 × 10⁴ mL⁻^1^, *B. cinerea* primarily penetrates hosts via ICs [7]. Given the dispersion pattern of conidia in the field, low concentrations are a common scenario, making ICs play an important role in the penetration process [5].

Despite the critical role of ICs in pathogenesis, their developmental mechanisms remain poorly understood, with limited research available. Due to a historical lack of attention to IC development, many studies focused on the pathogenicity genes of *B. cinerea* have not analyzed the phenotype of mutants related to IC formation. Instead, we can only rely on detailed descriptions of pathogenicity in the literature to infer whether relevant genes are involved in IC development.

The formation of ICs requires two types of signals. One is the developmental state (or developmental signal), where only mature hyphae have the potential to develop into ICs. Germ tubes from conidia lack this ability, and must further branch and grow into mature hyphae before developing ICs. The second signal is a hard interface, such as the surface of plant leaves or fruits. In the laboratory, hydrophobic plastic covers and hydrophilic glass slides can both induce the formation of ICs from mature hyphae when they contact these hard surfaces [8]. *B. cinerea* likely perceives host surface signals through surface receptors or proteins, such as G-protein-coupled receptors or the signaling mucin Msb2 [9], which then transduce the signals into hyphal cells. Intracellular signaling is dependent on two classical signaling pathways: the cAMP pathway and the MAPK pathway. Mutants with deficient key components of the cAMP pathway, such as Δ*bcg3* (G-protein deletion strain) and Δ*bac* (adenylate cyclase deletion strain), lose their ability to invade the host [10,11,12]. Similarly, mutants with knockouts of key MAPK signaling components, such as Ste11 (MAPKKK), Ste7 (MAPKK), bmp1 (MAPK), Ste50 (MAP kinase adaptor), and Ste12 (MAPK downstream transcription factor), also lose host invasion ability [11,13]. Additionally, studies have shown that reactive oxygen species (ROS) also regulate the development of ICs. Superoxide anions (O₂^−^) produced by NADPH oxidase are involved in this regulation, as NADPH oxidase mutants (Δ*noxA*, Δ*noxD*) and their regulatory protein mutant Δ*noxR* cannot form ICs [14]. Recently, our team found that knocking out the ribosomal 60S subunit assembly protein Nop53 causes *B. cinerea* to completely lose the ability to form ICs, suggesting that the normal functioning of the protein synthesis machinery is crucial for IC formation [15]. By knocking out the core septin Sep4 in both *B. cinerea* and the rice blast fungus, we identified that Sep4 regulates the initial development of infection structures such as appressoria and ICs [16]. Preliminary studies indicated that Sep4 functions downstream of cAMP, ROS, and other signaling pathways, to mediate the initiation of infection structure development [16]. Our recent work showed that deletion of the histone H3 demethylase Jar1 disrupts the subcellular localization of Sep4, and these mutants completely lose the ability to form ICs, indicating that Sep4’s function is regulated at the DNA level [17].

To thoroughly unravel the mechanisms behind IC development in *B. cinerea*, we need to identify more genes involved in this process. The most effective way to achieve this goal is the forward genetics approach [18,19]. By creating a high-throughput mutant library with defects in IC development, we can conduct large-scale identification of associated genes and analyze their functions and mechanisms. This would provide new insights and potential entry points for a comprehensive understanding of the formation and function of ICs, the key infection structure of *B. cinerea*. In this study, we used the *Agrobacterium tumefaciens*-mediated transformation (ATMT) method to transform *B. cinerea* and established a large T-DNA-tagged population containing 14,000 transformants. Through phenotype analysis, we identified 161 mutants with defects in IC development, establishing a large mutant library. We analyzed the T-DNA insertion site of a mutant that was unable to form ICs and identified the tagged gene as *EXO70*, which encodes a component of the exocyst. Upon knocking out this gene, we found that the deletion mutant lost the ability to form ICs. This result validates the effectiveness of the mutant library we established. The IC development-deficient mutant library we have reported here provides a solid foundation for further large-scale identification of genes involved in IC development and for unraveling the developmental mechanisms of this large infection structure.

## 2. Materials and Methods

### 2.1. Fungal Strains and Culture Conditions

*B. cinerea* strains used here are listed in Table 1. The wild-type (WT) strain B05.10 and its derived strains, including random T-DNA insertional transformants, the knockout mutant ∆*exo70*, and the complemented strain ∆*exo70*-C, were cultured on potato dextrose agar (PDA) plates, as previously described [16]. Strains were stored on PDA in Eppendorf tubes at 4 °C or covered with 20% glycerol at −20 °C.

### 2.2. Creation of T-DNA Randomly Insertional Transformant Population of B. cinerea

For creating the T-DNA randomly insertional transformant population of *B. cinerea*, the binary vector pBHt2 (Table 2), was used to transform the WT strain B05.10 via the *Agrobacterium tumefaciens*-mediated transformation (ATMT) method, as described [16], with PDA plates containing 100 mg/L hygromycin as the selection condition.

### 2.3. Screening Method of IC Formation-Deficient Mutants

For IC induction, fresh mycelial plugs taken from edges of the colony were inoculated on glass slides, cultured at 20 °C, and observed at 20–40 h. Each strain was subjected to three biological replicates, with three technical replicates per replicate. For each sample, six random fields were observed, and transformants with an IC count less than 20% of the wild-type number, or those unable to form mature ICs, were categorized as IC formation-deficient mutants. For quantitative analysis, each strain was evaluated according to the IC rank based on the percentage of IC number to the wild type: 5 (80–100%), 4 (50–80%), 3 (20–50%; or unmature), 2 (10–20%), 1 (<10%), 0 (0%). Conidia were also observed for IC development, with 10 μL conidial suspensions [10^5^ conidia/mL in 1/2 potato dextrose broth (PDB)] inoculated on glass slides.

### 2.4. Identification of T-DNA-Tagged Genes

Thermal asymmetric interlaced-PCR (TAIL-PCR) was employed to identify T-DNA insertion sites in the genome of mutants with the method described previously [21,23,24]. Briefly, three specific nested primers annealing to the T-DNA right border and an arbitrary primer (Table 3) were used to amplify the flank sequence of inserted T-DNA. By sequencing and the following sequence analysis, the T-DNA insertion sites in the mutant genomes were identified, and T-DNA-tagged genes were confirmed, consequently.

### 2.5. Gene Knockout and Genetic Complementation

Vectors and primers used in this study are listed in Table 2 and Table 3, respectively. The gene replacement method was used for *EXO70* knockout. The vector pXEH, containing the hygromycin resistance gene *HPH* [16], was used to construct the knockout vector. The 5’- and 3’- homologous flanks of *EXO70* were amplified with Q5 high-fidelity DNA polymerase (NEB, Ipswich, MA, USA), and cloned into pXEH in the upstream and downstream of *HPH*, respectively. The resultant vector pEXO-KO was transformed into *B. cinerea* via the ATMT method. The transformants were selected on PDA plates supplemented with 100 mg/L hygromycin.

The vector pSULPHGFP [22], resistant to chlorimuron ethyl, was used to generate the complemented strain ∆*exo70*-C. The full fragment of *EXO70* was amplified by PCR and cloned into pSULPHGFP to generate the complemented vector pEXO-C, which was then transformed into ∆*exo70*. The transformants were selected on DCM plates containing 100 mg/L chlorimuron ethyl, as previously described [25].

The *EXO70* deletion mutants and complemented strains were further confirmed by quantitative reverse transcription PCR (qRT-PCR) [26]. DNA and RNA were extracted as previously described [27,28].

### 2.6. Growth and Conidiation Assays

The growth and conidiation assays of *B. cinerea* were performed as previously described [16]. Briefly, the growth was determined by measuring the colony diameter of the cultures on PDA at 72 h. For quantitative analysis, each mutant strain was evaluated according to the growth rank based on the percentage of colony diameter to the wild type: 5 (80–100%), 4 (50–80%), 3 (20–50%), 2 (10–20%), 1 (<10%). For conidiation, PDA cultures were induced with light at 20–25 °C and conidial number per plate was calculated using a hemocytometer at 12 days post inoculation (DPI).

### 2.7. Pathogenicity Assays

Pathogenicity was assayed as described previously [16]. Briefly, mycelial plugs (5 mm in diameter) were inoculated on detached green bean leaves, incubated in plastic containers with high humidity at 20–25 °C, and observed at 48 h post inoculation (HPI). Plugs derived from the wild-type strain B05.10 or blank plates were used as controls.

### 2.8. Statistical Analysis

All the quantitative data resulted from triplicate experiments, independently. The significance of the data was assessed using the Student’s *t*-tests, and a *p*-value lower than 0.05 was considered to be statistically significant.

## 3. Results

### 3.1. Establishment of a T-DNA-Tagged Population Using the ATMT Method

*B. cinerea* is a highly successful crop killer, but its IC developmental mechanisms remain poorly understood. To identify genes involved in IC formation on a large scale, we employed a forward genetics approach and used the ATMT method to transform *B. cinerea*. After screening with hygromycin, we established a large T-DNA-tagged population containing 14,000 transformants. Each transformant in this population contains at least one randomly inserted T-DNA in its genome. The insertion of T-DNA may inactivate native genes (e.g., by inserting into coding regions) or reduce their expression levels (e.g., by inserting into promoter regions or UTRs) [29]. Therefore, this T-DNA-tagged population lays the foundation for screening mutants with defects in IC development.

### 3.2. Screening of IC Formation-Deficient Mutants

We next evaluated the IC development phenotypes of the T-DNA-tagged population mentioned above. We used a simple and effective method to induce ICs by placing mycelial plugs onto glass slides. Under this induction condition, the wild-type strain B05.10 typically forms a large number of ICs at 20 h, with more than 20 ICs per 10 × 10 microscopic field. Considering that some mutants may exhibit slower growth, which could theoretically delay the formation of ICs, we doubled the induction time to 40 h. Additionally, since there will be variability in the IC formation phenotype between replicates, we refined our screening criteria to better identify mutants with defects in IC development. For each tested strain, we counted the number of ICs per microscope field under 10 × 10 magnification. Strains with fewer than 20% of the wild-type IC number were classified as mutants with deficient IC formation. Using this stringent screening approach, we identified 161 mutants with significantly reduced IC development from the T-DNA-tagged population (Table 4), thereby establishing a refined mutant library of *B. cinerea* with defects in IC development.

### 3.3. Phenotype Analysis of B. cinerea IC Development-Deficient Mutants

**Figure 1:**
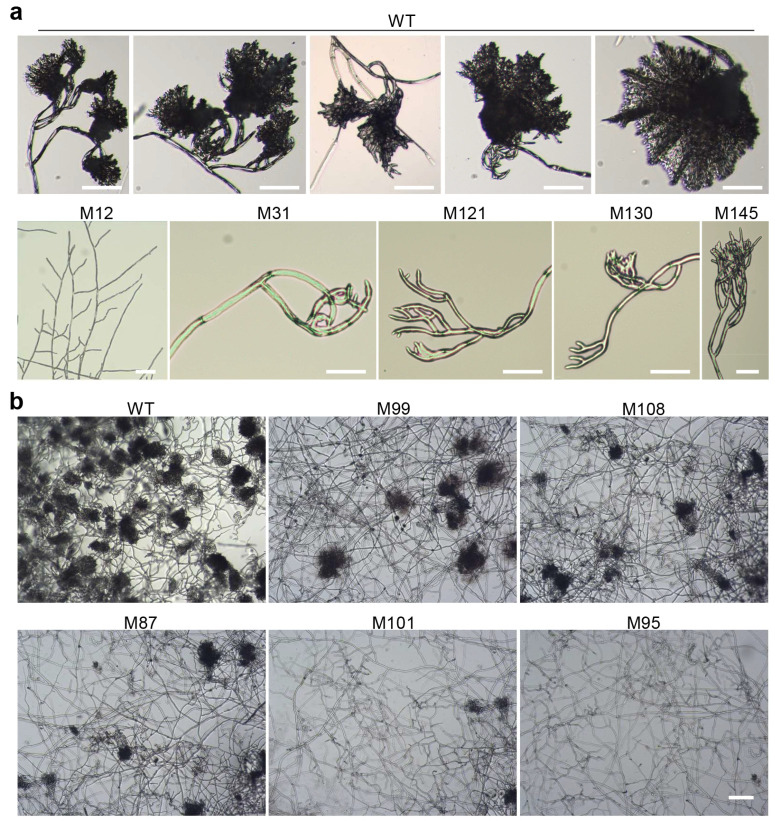
Phenotypes of several representative infection cushion (IC) development-deficient mutants of *B. cinerea*. (**a**) Mutants that do not form ICs or whose ICs cannot mature. Mycelial plugs of the tested strains were inoculated on glass slides to induce IC development and observed at 40 h post inoculation (HPI). Bar = 50 μm. (**b**) Mutants with a significantly reduced number of ICs. Conidial suspensions (10^5^ conidia/mL in 1/2 PDB) of the tested strains were inoculated on glass slides to induce IC development and observed at 40 HPI. Bar = 100 μm. WT, the wild-type strain B05.10. Other strains are selected typical T-DNA-tagged mutants.

We conducted a detailed phenotype analysis of the IC development-deficient mutants (Table 4 and Table 5). Our results showed that some mutants completely lost the ability to form ICs, while others had impaired IC formation and could only develop immature primary ICs (Figure 1a). Additionally, some mutants exhibited a significant reduction in the number of ICs formed (Figure 1b).

Among the 161 mutants, 86 were unable to form ICs. Over half of these strains (54 mutants) showed significantly slower hyphal growth, and 37 of 54 strains exhibited very slow growth. This indicates that genes important for hyphal growth often play crucial roles in IC development, particularly in the initiation of IC formation, likely because these mutants failed to form ICs from the beginning. When these genes were inactivated by T-DNA insertion, the mutants lost the ability to initiate IC development, resulting in the failure to form ICs and a marked reduction in hyphal growth. Another 32 mutants grew normally, suggesting that the mutated genes are specifically required for IC development but are not essential for hyphal growth.

Among the 161 mutants, 29 showed a significant reduction in the number of ICs formed (Table 5), with fewer than 20% of the wild-type number. Approximately one-third of these mutants (9 strains) had slightly slower growth, while the remaining two-thirds (20 strains) grew normally. There were also 44 mutants in the library that were hampered in IC formation and did not form mature ICs. Most of these mutants grew normally.

To analyze the relationship between IC development and conidiation, we randomly selected 10 mutants with defects in IC development (M4, M6, M15, M21, M22, M24, M32, M33, M50, M59, M135) and examined their conidiation ability. We found that only two mutants (M4 and M135) were able to produce conidia, while the other eight mutants were unable to produce conidia. This suggests that the processes of IC formation and conidiation are highly overlapping, and many genes are involved in both developmental processes.

### 3.4. Functional Identification of a Key Regulatory Gene for IC Development in B. cinerea

From the established mutant library, we randomly selected a mutant, M12, which was unable to form ICs for further investigation. Using TAIL-PCR amplification and sequence analysis, we found that the T-DNA was inserted into the 5′ UTR region of the *EXO70* gene in this mutant. *EXO70* encodes a core component of the exocyst complex, which is responsible for the transport of secretory vesicles to the apical membrane of hyphae, where these vesicles fuse with the membrane to release their contents or become incorporated into the membrane, playing an essential role in processes such as hyphal growth and development.

**Figure 2:**
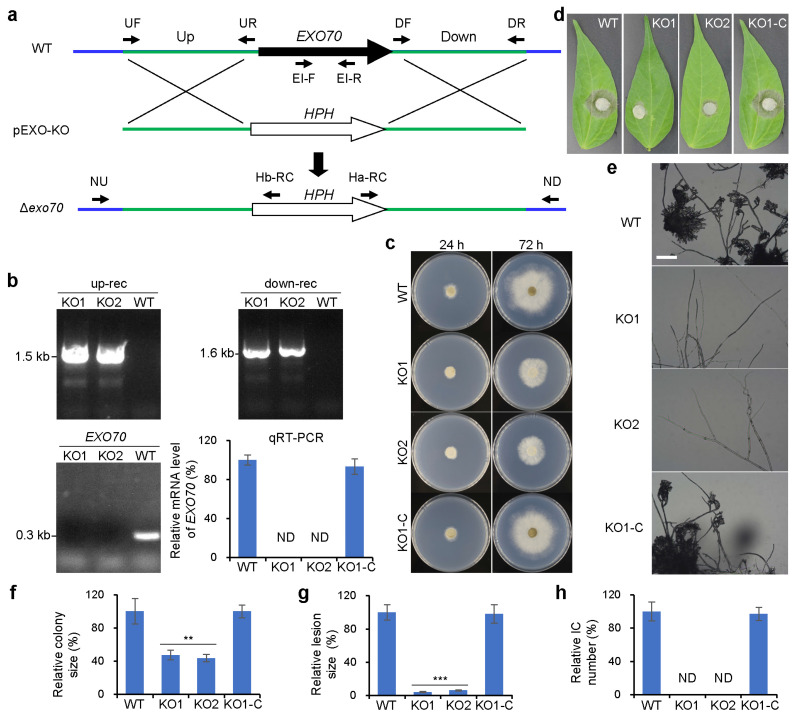
*EXO70* is essential for IC development of *B. cinerea*. (**a**) Strategy for generation of *EXO70* knockout strain Δ*exo70* via gene replacement approach. WT, the wild-type strain B05.10; pEXO-KO, *EXO70* knockout vector. *HPH*, the hygromycin resistance gene. Blue lines and green lines indicate genomic sequences and *EXO70* homologous flanks, respectively. (**b**) Molecular identifications of knockout mutants Δ*exo70* (KO1 and KO2) and the complemented strain Δ*exo70*-C (KO1-C). PCR amplifications were used for detecting *HPH* upstream recombination (up-rec) with primers NU/Hb-RC, downstream recombination (down-rec) with primers Ha-RC/ND, and *EXO70* loss with primers EI-F/EI-R, respectively. Relative *EXO70* expression levels in the indicated strains were determined by quantitative reverse transcription PCR (qRT-PCR). (**c**) Colony of tested strains cultured on PDA. (**d**) Pathogenicity assays with mycelial plugs inoculated on detached green-bean leaves at 48 HPI. (**e**) IC development on glass slides at 40 HPI. Bar = 100 μm. (**f**) Quantification of the colony sizes at 72 h shown in (**c**). (**g**) Quantification of the lesion sizes shown in (**d**). (**h**) Quantification of the IC numbers shown in (**e**). ND, not detected. Data represent means ± standard deviations (SD) from at least three independent experiments. **, ***, significance at *p* < 0.01, 0.001, respectively.

In *B. cinerea*, *EXO70* has been reported to be involved in growth, conidiation, sclerotia production, and virulence [30]. However, whether it plays a role in IC development remains unclear. To confirm whether the inability of mutant M12 to form ICs was due to the T-DNA insertion in the 5’ UTR region of *EXO70*, we created two independent *EXO70* knockouts, KO1 and KO2, in the wild-type strain B05.10 (Figure 2a,b).

Our data showed that *EXO70* is involved in both growth (Figure 2c,f) and pathogenicity (Figure 2d,g), which is consistent with previous reports [30]. Similar to the phenotype of the T-DNA insertion mutant M12, we found that the *EXO70* knockout strains completely lost the ability to form ICs (Figure 2e,h). When induced on glass slides for 48 h, the mutant was unable to initiate IC formation. Given that the *EXO70* knockout mutants exhibited significantly slower growth, we extended the observation time to 96 h, but the mutants still failed to initiate IC formation. In contrast, the complemented strain fully restored IC formation, showing no significant difference from the wild-type strain.

These results indicate that *EXO70* is a key regulatory factor for IC formation in *B. cinerea* and plays a critical role in the initiation of IC development, likely because the knockout mutants failed to form ICs from the outset.

## 4. Discussion

The development of IC in *B. cinerea* plays a pivotal role in the fungus’s ability to breach plant physical defenses and establish infection. Despite its importance in pathogenesis, the molecular mechanisms that regulate IC formation have been poorly understood. In this study, we established a comprehensive T-DNA insertion mutant library to identify genes involved in IC development. By screening 14,000 transformants, we identified 161 mutants with defective IC formation, providing a valuable resource for dissecting the genetic pathways underlying this critical infection process.

Our screening results revealed a range of phenotypes among the mutants. Some mutants completely failed to form ICs, while others displayed partial defects, such as the formation of immature or fewer ICs. Interestingly, many of the mutants with severe defects in IC development also exhibited slow or impaired hyphal growth, indicating that the genes required for IC formation are often involved in fundamental processes such as hyphal growth and development. This observation aligns with previous studies showing that signaling pathways and cellular processes regulating growth, such as the cAMP and MAPK pathways, also influence host-penetration ability [10,13]. Additionally, the development of the IC is strongly correlated with conidiation. Mutants with severe defects in the IC often show a significant reduction in their ability to produce conidia. Their pathogenicity phenotype is found to be complex, and a separate, more comprehensive follow-up study would be needed. *EXO70* serves as a typical example. We found that the *EXO70* deletion mutants are unable to form ICs. Previous reports and our data have indicated that mutants lacking this gene have difficulty forming conidia. It is also noteworthy that some mutants grew normally but exhibited specific defects in IC formation, suggesting that distinct regulatory mechanisms may control these processes independently.

A key finding of our study was the identification of *EXO70* as a critical regulator of IC formation. *EXO70* encodes a component of the exocyst complex, which is involved in vesicle trafficking and membrane fusion, processes essential for hyphal growth and cellular development. Our results show that the disruption of *EXO70* completely abolished IC formation, confirming its role in this key aspect of pathogenicity. The observation that the *EXO70* knockout strains also exhibited impaired growth further underscores the importance of vesicle trafficking in both vegetative growth and the development of infection structures. These findings are consistent with the broader role of *EXO70* in other fungal processes, such as host penetration, virulence, and secretion of effectors [31,32,33].

The involvement of *EXO70* in IC formation adds new knowledge to our understanding of the cellular machinery underlying fungal pathogenesis. Given its essential role in the trafficking of vesicles to the apical membrane, *EXO70* likely mediates the delivery of critical proteins and membrane components necessary for the morphogenesis of infection structures. This suggests that IC development may rely on tightly regulated exocytosis and membrane dynamics, processes that could be targeted for the development of new antifungal strategies.

In addition to *EXO70*, our mutant library provides a wealth of other candidate genes that may regulate IC development. The diversity of phenotypes observed in the mutants suggests that multiple signaling pathways, including those involved in growth, environmental sensing, and cellular stress responses, contribute to the formation of ICs. Future studies focused on the functional characterization of these genes will help uncover the intricate regulatory networks that coordinate infection structure development in *B. cinerea*.

In conclusion, the establishment of the IC-deficient mutant library and the identification of *EXO70* as a key regulatory gene for IC development represent significant advances in our understanding of *B. cinerea* pathogenesis. This study lays the groundwork for future genetic and biochemical investigations into the molecular mechanisms governing IC formation, and will offer new targets for controlling the devastating impact of *B. cinerea* on crop production.

## Figures and Tables

**Table 1:** Strains of *Botrytis cinerea* used in this study.

Strain	Description	Source
B05.10	Wild-type strain	[20]
IC formation-deficient mutants ^1^	Random T-DNA insertional mutants derived from B05.10	This study
Δ*EXO70*	B05.10 with *EXO70* knocked out, Δ*EXO70*::*HPH*	This study
Δ*EXO70*-C	The ectopic complementary strain of Δ*EXO70*	This study

^1^ IC, infection cushion.

**Table 2:** Plasmid vectors used in this study.

Vector	Description	Source
pBHt2	Binary vector used for generation of T-DNA insertional transformants	[21]
pXEH	Binary vector used for knockout of fungal genes, containing *HPH* gene within its T-DNA region; Km^R^	[16]
pSULPHGFP	Binary vector containing *ILV1* gene (resistance to chlorimuron ethyl) within its T-DNA region; Km^R^	[22]
pEXO-KO	Constructed from pXEH, for knockout of *EXO70*	This study
pEXO-C	Constructed from pSULPHGFP, for genetic complementation of Δ*EXO70*	This study

**Table 3:** Primers used in this study.

Primer Name	Primer Sequence (5′-3′)
For thermal asymmetric interlaced-PCR (TAIL-PCR)	
RB1	GGCACTGGCCGTCGTTTTACAAC
RB2	AACGTCGTGACTGGGAAAACCCT
RB3	CCCTTCCCAACAGTTGCGCA
AD1	TGWGNAGWANCASAGA
For construction of *EXO70* knockout vector	
EXO70-UF	CTCGAGTGTGGGAAATGTGGGATG
EXO70-UR	GAATTCGCACGCTAGACCTAATGC
EXO70-DF	TCTAGACCTGTGGGTGAGACGAGA
EXO70-DR	AAGCTTAATGCGAAATGCGAAACT
For screening *EXO70* knockout strains	
NU	CGACATAACGAAGTGGGATT
ND	AACCCGCAACAACCATAAAC
Ha-RC	ATGATGCAGCTTGGGCGCA
Hb-RC	ACAGACGTCGCGGTGAGTTCA
EI-F	CCGCCACGGTTCCAATGAC
EI-R	TTCCCAAAGCAGGCTTACCA
For genetic complementation of Δ*EXO70*	
EXO70-CF	TACCGTCGACGACATAACGAAGTGGGATTG
EXO70-CR	CCGCTCTAGAATCACTTATTCACCCGTCCC
For qRT-PCR analysis of *Actin*	
ACT-F	CATGGCTGGTCGTGATTTGA
ACT-R	GAGGATTGACTGGCGGTTTG

**Table 4:** Infection cushion (IC) development-deficient mutants of *B. cinerea* derived from T-DNA random insertions.

Mutant	IC ^1^	Growth ^2^	Mutant	IC	Growth	Mutant	IC	Growth
M1	0	5	M55	0	2	M109	1	4
M2	0	5	M56	0	2	M110	2	4
M3	0	5	M57	0	2	M111	2	4
M4	0	5	M58	0	3	M112	2	4
M5	0	5	M59	0	1	M113	2	4
M6	0	5	M60	0	3	M114	2	4
M7	0	5	M61	0	3	M115	2	4
M8	0	5	M62	0	3	M116	3	5
M9	0	5	M63	0	3	M117	3	5
M10	0	5	M64	0	3	M118	2	5
M11	0	5	M65	0	2	M119	3	5
M12	0	5	M66	0	2	M120	3	5
M13	0	5	M67	0	2	M121	3	5
M14	0	5	M68	0	2	M122	3	5
M15	0	5	M69	0	2	M123	3	5
M16	0	5	M70	0	2	M124	3	5
M17	0	5	M71	0	2	M125	3	5
M18	0	5	M72	0	1	M126	3	5
M19	0	5	M73	0	2	M127	2	5
M20	0	5	M74	0	2	M128	3	5
M21	0	5	M75	0	2	M129	3	5
M22	0	5	M76	0	2	M130	3	5
M23	0	5	M77	0	2	M131	3	5
M24	0	5	M78	0	2	M132	3	5
M25	0	5	M79	0	2	M133	3	5
M26	0	5	M80	0	2	M134	3	5
M27	0	5	M81	0	2	M135	3	5
M28	0	5	M82	0	1	M136	3	5
M29	0	5	M83	0	2	M137	3	5
M30	0	5	M84	0	2	M138	3	5
M31	0	5	M85	0	2	M139	3	5
M32	0	5	M86	0	2	M140	3	5
M33	0	4	M87	1	5	M141	2	5
M34	0	4	M88	1	5	M142	3	5
M35	0	4	M89	2	5	M143	3	5
M36	0	4	M90	1	5	M144	3	5
M37	0	4	M91	1	5	M145	3	5
M38	0	4	M92	1	5	M146	3	5
M39	0	4	M93	2	5	M147	2	5
M40	0	4	M94	2	5	M148	3	5
M41	0	4	M95	1	5	M149	3	5
M42	0	4	M96	1	5	M150	3	5
M43	0	4	M97	1	5	M151	3	4
M44	0	4	M98	1	5	M152	3	4
M45	0	4	M99	1	5	M153	3	4
M46	0	4	M100	2	5	M154	2	4
M47	0	4	M101	1	5	M155	3	4
M48	0	4	M102	1	5	M156	3	4
M49	0	4	M103	1	5	M157	2	2
M50	0	3	M104	1	5	M158	3	2
M51	0	2	M105	1	5	M159	3	3
M52	0	3	M106	1	5	M160	3	5
M53	0	3	M107	1	4	M161	3	5
M54	0	3	M108	1	4	WT ^3^	5	5

^1^ Each strain we evaluated according to the IC rank, based on the percentage of IC number to the wild type; ^2^ hyphal growth was evaluated according to the growth rank based on the percentage of colony diameter to the wild type. The rating criteria are detailed in the Materials and Methods section. ^3^ The wild-type strain B05.10.

**Table 5:** Statistics of the IC developmental phenotypes of the mutant library.

**IC phenotype**	none			rare ^1^		unmature ^2^			slow
**hyphal growth**	normal	slow slightly	very slow	normal	slow slightly	normal	slow slightly	very slow	normal
**mutant number**	32	17	37	20	9	35	6	3	2
**total number**	86			29		44			2

^1^ Mutants with an IC count less than 20% of the wild-type number; ^2^ mutants with an IC count less than 50% of the wild-type number, most of which were unmature.

## Data Availability

Data are contained within the article.
